# Use of Factorial Designs to Reduce Stability Studies for Parenteral Drug Products: Determination of Factor Effects via Accelerated Stability Data Analysis

**DOI:** 10.3390/pharmaceutics17081067

**Published:** 2025-08-18

**Authors:** Lara Pavčnik, Simona Bohanec, Tina Trdan Lušin, Robert Roškar

**Affiliations:** 1 Sandoz Development Center Slovenia, Lek Pharmaceuticals d.d., Verovškova 57, SI-1526 Ljubljana, Slovenia; lara.pavcnik@sandoz.com (L.P.); simona.bohanec@sandoz.com (S.B.); tina.trdan_lusin@sandoz.com (T.T.L.); 2 Department of Biopharmaceutics and Pharmacokinetic, Faculty of Pharmacy, University of Ljubljana, SI-1000 Ljubljana, Slovenia

**Keywords:** factorial designs, regression analysis, ICH Q1D, stability study, degradation kinetics, parenteral dosage forms

## Abstract

**Objectives:** This study explores the potential of factorial analysis as an alternative strategy for optimizing stability study designs for registration batches—an approach not currently addressed in ICH Q1D, which focuses solely on bracketing and matrixing. The objective is to assess the reliability of stability designs reduced based on factorial analysis and the extent to which long-term stability testing can be reduced using this approach. **Methods:** To determine the feasibility of applying factorial analysis for stability study design reduction while preserving the reliability of stability assessments, three parenteral dosage forms were selected. Stability data under both accelerated and long-term storage conditions were analyzed. Factorial analysis was applied to the accelerated data to identify critical factors influencing stability (e.g., filling volume, orientation). Based on these findings, long-term study designs were strategically reduced, and the validity of these reductions was confirmed through regression analysis of long-term data. **Results:** Factorial analysis revealed key factors significantly affecting stability, including batch, orientation, filling volume, and drug substance supplier. The analysis identified the worst-case scenarios and, based on this, proposed a drastic reduction in the long-term stability study designs for three tested parenteral drug products. The regression analysis results confirmed the usefulness of factorial analysis for the reduction of long-term stability testing of tested parenteral drug products for at least 50%. **Conclusions:** This study demonstrates that factorial analysis of accelerated stability data is a valuable tool for optimizing long-term stability study designs for parenteral pharmaceutical dosage forms. The findings suggest that this approach could complement existing ICH Q1D strategies, offering the pharmaceutical industry a scientifically sound method to streamline stability programs, reduce costs, and accelerate development timelines while maintaining product quality, safety, and efficacy.

## 1. Introduction

Stability studies of pharmaceutical products are a critical aspect of pharmaceutical development and manufacturing. Especially for parenteral drug products, which are sterile preparations intended for administration by injection, infusion, or implantation into the human body. Since parenteral products are introduced by bypassing the body’s most important protective barriers, the skin and mucous membranes, they must be “essentially” free of physical, chemical, and biological impurities. Stability studies should include testing of those attributes of the drug product that are susceptible to change during storage and are likely to influence quality, safety, and efficacy. Parenteral dosage forms, like all other dosage forms, need to maintain both chemical and physical stability throughout the shelf life of the product. Moreover, microbiological evaluation is an additional stability requirement (i.e., maintenance of sterility and apyrogenicity). Also, the presence of particulate matter represents potential safety risks to patients; therefore, this parameter should be monitored during a stability study [1,2,3]. These studies determine the shelf life and storage conditions for pharmaceutical drug products, ensure their safety and efficacy throughout the lifecycle, and are required for regulatory approval. Stability studies generally last for several years and, therefore, require a considerable amount of time and resources, which contributes to the high costs. According to the International Council for Harmonization (ICH) guideline Q1A (R2), the stability study of drug products usually involves testing at various time points, including long-term (0, 3, 6, 9, 12, 18, 24 months), intermediate (0, 3, 6, 9, 12 months), and accelerated (0, 3, 6 months) conditions. To meet the requirements, three registration batches should be manufactured for each strength and included in the stability study. For parenteral dosage forms, the stability study must also be conducted in two orientations [1]. However, in line with the reduced stability principles outlined in ICH Q1D (Bracketing and Matrixing Designs for Stability Testing of New Drug Substances and Products), bracketing and matrixing designs can be applied. Bracketing design is suitable when a drug product involves three or more strengths. In this approach, the stability study is only performed on the extreme strengths. On the other hand, matrixing design is defined as a statistical design of a stability schedule so that only a fraction of the total number of samples is tested at a given sample time point. The design assumes that the stability of each subset of samples tested represents the stability of all samples at a given time point [4]. Nevertheless, for both bracketing and matrixing designs, it is essential to conduct a long-term stability study for all planned batches until the end of the product’s shelf life. In April 2025, a draft revision of the ICH Q1 guideline was issued. However, no significant changes were introduced regarding the principles of reduced stability testing. The concepts of bracketing and matrixing remain consistent with the previously established guidance, reaffirming their applicability under defined conditions and emphasizing the importance of scientific justification and regulatory acceptance [5].

Another approach to reduce stability studies, which is currently neither included in the stability guidelines (current and draft) nor used in pharmaceutical development to optimize stability testing of registration batches, is the application of factorial analysis using accelerated data. In this method, the accelerated stability data are analyzed to determine the worst-case scenario. Based on this analysis, the testing under long-term conditions is then continued for the combination of factors that determine the shelf life of the drug product. The discussion about this approach initially took place through presentations and round table discussions at stability conferences [6]. Feedback from industry has been positive, while feedback from the regulatory authorities on the strategy for additional reduction of the stability study has been limited.

Factorial analysis, also known as factorial design, is a statistical method used to study the effects and interactions of multiple factors on a response variable. It allows understanding of how different factors, such as drug formulation, dosage, and process parameters, influence the response of a drug. In factorial analysis, a factorial design is created by systematically varying each factor at different levels and then conducting experiments to measure the response variable under each combination of factor levels. By analyzing the data from these experiments, the main effects of each factor and interactions between factors can be determined [7,8].

Factorial analysis in pharmaceutical drug development offers several advantages. One major advantage is the ability to investigate multiple factors simultaneously, instead of analyzing them individually. This comprehensive approach enables the identification of the most influential factors and their interactions and provides valuable insights. Secondly, the use of factorial analysis can optimize drug development processes. It enables the identification of critical process parameters that should be controlled during manufacturing to achieve the desired drug properties. This understanding helps to optimally formulate the medicine and determine the most favorable manufacturing conditions. Finally, the application of factorial analysis facilitates the efficient identification of optimal drug formulations, dosages, and manufacturing parameters. By using factorial analysis, drug development processes can be improved and production costs reduced. Overall, factorial analysis is a tool with potential for broader application in pharmaceutical development [9,10].

Several articles are available on the application of factorial analysis for stability evaluation, which relate to the evaluation of packaging [11,12], process, and formulation parameters [13] for solid dosage forms. These publications focus on the application of factorial analysis for the purpose of development stability—supporting the selection of appropriate packaging, process, and formulation parameters for registration batches. However, no data are available on the application of factorial designs to parenteral dosage forms for the purpose of registration stability. Traditional stability study reductions, such as bracketing and matrixing, have primarily been evaluated on solid dosage forms in the past [14,15,16,17]. Studies by Oliva et al. and Pavčnik et al. have explored the application of matrixing design specifically for parenteral pharmaceutical dosage forms [18,19]. Additionally, Pavčnik et al. also investigated the reduction of long-term stability studies using the ASAP approach [20].

The objectives of this study were (1) to explore factorial analysis as another approach to reduce stability studies of registration batches, which is not currently addressed in ICH Q1D; (2) to determine the magnitude/significance of the influence of individual factors based on the results of the factorial analysis and set a significance limit; and (3) to evaluate the extent to which long-term stability studies can be reduced using this approach. To evaluate the suitability of the factorial analysis approach for optimizing stability study design while maintaining a reliable assessment of product stability, three parenteral dosage forms were selected and examined their stability data under both accelerated and long-term storage conditions.

## 2. Materials and Methods

### 2.1. Drug Product

Three parenteral pharmaceutical products developed and manufactured by Sandoz were evaluated. The iron product is an aqueous colloidal dispersion for injection/infusion containing 50 mg of iron/1 mL. Three batches of 1000 mg/20 mL were produced and packed into clear, colorless type I glass vials with bromobutyl rubber stoppers and aluminum crimp caps with plastic flip-off. Pemetrexed is available as a solution for infusion into a vein; 1 mL of the product contains pemetrexed sodium, equivalent to 25 mg of pemetrexed. It was produced in three filling volumes, 100 mg/4 mL, 500 mg/20 mL, and 1000 mg/40 mL, and packed into clear, colorless type I glass vials with bromobutyl rubber stoppers and aluminum crimp caps with light blue plastic flip-off. Sugammadex is a solution for injection administered intravenously as a single bolus injection. A 1 mL sample of the product contains sugammadex sodium, equivalent to 100 mg sugammadex. It was produced in two filling volumes, 200 mg/2 mL and 500 mg/5 mL, using active pharmaceutical ingredients (API) from different manufacturers (API1 and API2) and packed into type I glass vials sealed with bromobutyl rubber stoppers with aluminum caps and flip-off seals. Structures of the examined compounds are shown in the Supplementary Materials (Figure S1).

### 2.2. Stability Study Design

For each pharmaceutical product and each filling volume, three different batches were produced and stored in stability chambers under different storage conditions. The long-term stability study was performed under conditions of 25 °C ± 2 °C/60% RH ± 5% RH. Following ICH Q1A (R2), the pull points of the stability study were 3, 6, 9, 12, 18, and 24 months. In addition, all batches were subjected to an accelerated stability study at 40 °C ± 2 °C/75% RH ± 5% RH for 6 months and tested at 0, 3, and 6 months [1]. Stability studies were conducted under all conditions in two orientations: upright and inverted/horizontal orientation. The design of the stability study is summarized in Table 1.

### 2.3. Tested Parameters and Analytical Methods

For all three parenteral dosage forms, chemical (assay—LC; degradation products—LC/UV-VIS), physical (appearance—USP; color—USP; pH—USP; osmolality—USP; visible particles—visual inspection; subvisible particles—USP), and microbiological (sterility—USP membrane filtration method; bacterial endotoxins—USP Gel Clot Limit test) parameters were evaluated throughout the entire shelf life, in accordance with stability testing guidelines [1,21]. Stability studies should include testing of those attributes of the drug product that are susceptible to change during storage—stability-indicating parameters. During stability testing, an increasing trend in degradation products (DP) was observed, and, therefore, the factorial analysis was performed for the mentioned parameter. The degradation profile of all three drug products consists of several degradation products. However, an increasing trend in specific degradation products was observed. For the parenteral drug product pemetrexed, the degradation product pemetrexed S-dimer (according to USP); for sugammadex, mono-S-oxo-sugammadex (chemical name); and for the iron product, degradation product 1 was investigated. All three degradation products are referred to as DP1 in this article. Structures of monitored degradation products are shown in the Supplementary Materials (Figure S2). Validated high-performance liquid chromatography (HPLC), ultra-high-performance liquid chromatography (UHPLC), and ultraviolet-visible spectroscopy (UV-VIS) methods were used to determine the degradation products. The chromatographic conditions for the analytical methods used are listed in the Supplementary Materials (Table S1).

### 2.4. Factorial Analysis

Three complete factorial designs were employed to assess the accelerated stability data of parenteral pharmaceutical products. Two-level factorial designs were utilized to determine whether factors or interactions between factors influence the response and to estimate the significance of this effect. These factorial analyses involved conducting all possible combinations of experiments at two levels for each of the n-factors considered. Typically, the number of required experiments is 2^n^, where n denotes the number of factors. Thus, an n-factor two-level design is known as a (full) 2^n^ two-level factorial design. Specifically, a two-factor two-level complete factorial design was utilized for the iron product, considering the factors batch and orientation (Table 2). For pemetrexed, which involves three factors (batch, orientation, and filling volume), a three-factor two-level complete factorial design was implemented (Table 3). In the case of sugammadex, a four-factor two-level complete factorial design was conducted, considering batch, orientation, filling volume, and API (Table 4) [7,22]. The factorial analysis was performed for each product using the results for degradation products that are presented in the Supplementary Materials (Table S2–S9).

The levels can be represented in different ways. A common method is to label one level as + and the other as −. The combinations of + and − characterize an experiment. The effect is then calculated by summing all + and − values [22].

### 2.5. Data Analysis

Data calculation for factorial designs was performed using Excel (Microsoft Corporation, Microsoft 365, Redmond, WA, USA). For example, the effect *A* for two-factor two-level design in Table 2 was calculated in line with equation below:

(1) $$Effect\;A=\left(-y_{1}-y_{2}+y_{3}+y_{4}\right)/2$$

For three-factor two-level design in Table 3, the effect *A* was calculated in line with equation below:

(2) $$Effect\;A=\left({-y}_{1}-y_{2}-y_{3}-y_{4}+y_{5}+y_{6}+y_{7}+y_{8}\right)/4$$

And for four-factor two-level design in Table 4, the effect *A* was calculated in line with equation below:

(3) $$Effect\;A=\left({-y}_{1}-y_{2}-y_{3}-y_{4}-y_{5}-y_{6}-y_{7}-y_{8}{+y}_{9}+y_{10}+y_{11}+y_{12}+y_{13}+y_{14}+y_{15}+y_{16}\right)/8$$

One example of the procedure of calculating the effects from raw numbers is presented in Supplementary Materials, Procedure S1.

To assess if the calculated effects for factors and their interactions are relevant, the significance limit (*D_i_*) was calculated with the equation below:

(4) $$\left|D_{i}\right|=S_{E}\times t_{df}^{0.05}$$

In the above equation, *S_E_* is the experimental error, *t*^0.05^ is the tabulated value for Student distribution at 95% confidence and at *df* degrees of freedom of two-tailed *t*-test. Experimental error, *S_E_*, is expressed by interactions as follows:

(5) $$S_{E}=\sqrt{\frac{1}{n_{d}}\sum_{j=1}^{n_{d}}{\left(D_{j}\right)}^{2}}$$

where *D_j_* is the effect of the *j*-th interaction and *n_d_* is the number of high-order interactions [7,22,23]. This method for calculating the significance limit is appropriate if at least five high-order interaction effects are available. If there are four or fewer high-order interactions, a more suitable approach for establishing the significance limit is through the variability observed during method validation [23].

In addition, the long-term stability data were statistically evaluated using regression analysis as described in ICH guidelines [1]. As three different batches were produced for each product and filling volume, an analysis of covariance (ANCOVA) was performed to assess the batch-to-batch variability. If the analysis showed that the batch-to-batch variability is low, the data could be combined into an overall estimate. For this purpose, appropriate statistical tests (e.g., p-values for a significance level of rejection of more than 0.25) were applied to the slopes of the regression lines and the zero-time intercepts for the individual batches [24]. The shelf life was then calculated considering the specification limits for each product. The data analysis was performed using Minitab^®^ 20.3 (Minitab Inc., State College, PA, USA). Furthermore, the shelf life was also calculated for reduced long-term data based on a factorial design. The obtained shelf life for full and reduced designs was compared. In addition, the accuracy of the reduced designs (M_j_) in comparison to the full design (M1) was evaluated by root-mean-square error (RMSE). RMSE was calculated for each reduced design (M_j_) using six (non-zero) time points (i) and predicted values from the regression (y-hat):

(6) $${RMSE}_{M_{j}vsM1}=\sqrt{\frac{1}{6}\times \sum_{i=1}^{6}{\left({\hat{y}}_{i}^{M_{j}}-{\hat{y}}_{i}^{M1}\right)}^{2}}$$

The threshold used for our data was established by considering the quantification limit of the specific analytical method as well as the set permitted method error at that concentration level. For LC methods, the threshold, determining that an RMSE of 0.01% or lower is considered acceptable, was established based on the findings in the article by Pavčnik et al. [19]. However, for UV-VIS, the threshold, determining that an RMSE of 0.02% or lower is considered acceptable, was set by considering the quantification limit 0.10% and the permitted method error of 20% at this low concentration level (0.10%).

## 3. Results

Three parenteral pharmaceutical products were tested under accelerated and long-term stability conditions described in Section 2.2. Chemical, physical, and microbiological parameters were evaluated for all parenteral drug products discussed in this study. Tests on quantitative parameters such as assay, pH, and osmolality remained within the specified limits for each drug product, and no stability trend was observed (specified limit for assay is 90.0–105.0% for pemetrexed and sugammadex and 97.0–105.0% for the iron product; the specified limits for pH are: 5.0–7.0 for the iron product, 7.0–9.0 for pemetrexed, and 7.0–8.0 for sugammadex, and the specified limit for osmolality is 300–500 mOsmol/kg for sugammadex and 250–420 mOsmol/kg for iron product). However, a noteworthy increase was observed in the levels of degradation products over time, suggesting that degradation products are the stability-indicating parameter. The results for degradation products are presented in the Supplementary Materials (Table S2–S9). To systematically evaluate the applicability of factorial analysis in the stability studies of the mentioned parenteral drug products, the results were structured into two main parts. First, factorial analysis was performed using the degradation products’ accelerated data for each drug product, identifying differences in the number and nature of extracted factors. This provided insight into potential dimensionality reductions in the long-term stability data. In the second part, these findings were validated using linear regression models, assessing predictive performance through RMSE and calculating shelf life estimates. This two-step approach ensures both exploratory and confirmatory perspectives are addressed.

In Table 5, Table 6 and Table 7, the calculated effects of each factor are denoted as En, where “n” represents the specific factor being examined.

### 3.1. Factorial Analysis

#### 3.1.1. Iron Product

A two-factor two-level complete factorial analysis was performed for the iron product. The following factors were considered: number of batches (three batches) and orientation (upright and horizontal). The effects for factors and their interactions were calculated using Equation (1) from Section 2.5 and are presented in Table 5. The values in bold are above the determined significance limit.

**Table 5:** Calculated effects for factors and their interactions based on 6 months of accelerated data for the iron product. The values in bold are above the determined significance limit of 0.02%.

	E_A_ *, %	E_B_, %	E_AB_, %
Batch (A): 1 (−), 3 (+) Orientation (B): → (−), ↑ (+)	**0.145**	**0.045**	**0.075**
Batch (A): 1 (−), 2 (+) Orientation (B): → (−), ↑ (+)	−0.010	−0.010	0.020
Batch (A): 2 (−), 3 (+) Orientation (B): → (−), ↑ (+)	**0.155**	**0.065**	**0.055**

* The calculated effects of each factor are denoted as En, where “n” represents the specific factor being examined.

As only three higher-order interactions (E_AB_) were determined, the significance limit was set by the variability from the method validation for parameter degradation product, which is 0.02%.

#### 3.1.2. Pemetrexed

For pemetrexed, the three-factor two-level complete factorial analysis was performed. The following factors were considered: number of batches (three batches), orientation (upright and inverted), and filling volume (100 mg/4 mL, 500 mg/20 mL, and 1000 mg/40 mL). The calculated effects for factors and their interactions are presented in Table 6. The values in bold are above the determined significance limit.

**Table 6:** Calculated effects for factors and their interactions from 6 months of accelerated data for pemetrexed. The values in bold are above the determined significance limit of 0.048%.

	E_A_ *, %	E_B_, %	E_C_, %	E_AB_, %	E_AC_, %	E_BC_, %	E_ABC_, %
Batch (A): 1 (−), 3 (+) Orientation (B): ↓ (−), ↑ (+) Fill volume (C): 4 mL (−), 40 mL (+)	−0.038	**−0.063**	**−0.458**	0.007	0.043	**0.058**	−0.013
Batch (A): 1 (−), 2 (+) Orientation (B): ↓ (−), ↑ (+) Fill volume (C): 4 mL (−), 40 mL (+)	−0.043	−0.033	**−0.453**	0.038	0.048	0.038	−0.033
Batch (A): 2 (−), 3 (+) Orientation (B): ↓ (−), ↑ (+) Fill volume (C): 4 mL (−), 40 mL (+)	0.005	−0.025	**−0.410**	−0.030	−0.005	0.025	0.020
Batch (A): 1 (−), 3 (+) Orientation (B): ↓ (−), ↑ (+) Fill volume (C): 4 mL (−), 20 mL (+)	−0.040	**−0.060**	**−0.415**	0.015	0.040	**0.060**	−0.005
Batch (A): 1 (−), 2 (+) Orientation (B): ↓ (−), ↑ (+) Fill volume (C): 4 mL (−), 20 mL (+)	−0.040	−0.040	**−0.405**	0.035	**0.050**	0.030	−0.035
Batch (A): 2 (−), 3 (+) Orientation (B): ↓ (−), ↑ (+) Fill volume (C): 4 mL (−), 20 mL (+)	0.000	−0.025	**−0.365**	−0.020	−0.010	0.025	0.030
Batch (A): 1 (−), 3 (+) Orientation (B): ↓ (−), ↑ (+) Fill volume (C): 20 mL (−), 40 mL (+)	0.003	−0.003	−0.043	0.002	0.003	−0.003	−0.007
Batch (A): 1 (−), 2 (+) Orientation (B): ↓ (−), ↑ (+) Fill volume (C): 20 mL (−), 40 mL (+)	0.007	−0.002	−0.048	0.002	−0.002	0.007	0.003
Batch (A): 2 (−), 3 (+) Orientation (B): ↓ (−), ↑ (+) Fill volume (C): 20 mL (−), 40 mL (+)	−0.005	0.000	−0.045	0.000	0.005	0.000	−0.010

* The calculated effects of each factor are denoted as En, where “n” represents the specific factor being examined.

The significance limit is 0.048%, which was calculated based on data in Table 6 and Equation (4). For the calculation, the effects of interaction between batch, orientation, and filling volume were considered (E_ABC_).

#### 3.1.3. Sugammadex

For sugammadex, the four-factor two-level complete factorial analysis was performed. The following factors were considered: number of batches (three batches), orientation (upright and horizontal), filling volume (200 mg/2 mL and 500 mg/5 mL), and API (two different APIs). The calculated effects for factors and their interactions are presented in Table 7. The values in bold are above the determined significance limit.

**Table 7:** Calculated effects for factors and their interactions from 6 months of accelerated data for sugammadex. The values in bold are above the determined significance limit of 0.039%.

	Batch (A): 1 (−), 3 (+) Orientation (B): → (−), ↑ (+) Fill Volume (C): 2 mL (−), 5 mL (+) API (D): 1 (−), 2 (+)	Batch (A): 1 (−), 2 (+) Orientation (B): → (−), ↑ (+) Fill Volume (C): 2 mL (−), 5 mL (+) API (D): 1 (−), 2 (+)	Batch (A): 2 (−), 3 (+) Orientation (B): → (−), ↑ (+) Fill Volume (C): 2 mL (−), 5 mL (+) API (D): 1 (−), 2 (+)
E_A_ *, %	0.0037	**−0.0588**	**0.0625**
E_B_, %	0.0062	0.0038	0.0050
E_C_, %	**−0.0438**	0.0013	−0.0125
E_D_, %	**−0.1563**	**−0.1038**	**−0.1250**
E_AB_, %	0.0012	−0.0012	0.0025
E_AC_, %	−0.0137	0.0313	**−0.0450**
E_AD_, %	−0.0213	0.0313	**−0.0525**
E_BC_, %	−0.0062	−0.0062	−0.0075
E_BD_, %	−0.0037	−0.0013	−0.0050
E_CD_, %	**−0.0587**	−0.0038	−0.0175
E_ABC_, %	−0.0013	−0.0012	0.0000
E_ABD_, %	−0.0037	−0.0013	−0.0025
E_ACD_, %	−0.0137	**0.0412**	**−0.0550**
E_BCD_, %	−0.0013	−0.0013	−0.0025
E_ABCD_, %	−0.0013	−0.0013	0.0000

* The calculated effects of each factor are denoted as En, where “n” represents the specific factor being examined.

The significance limit is 0.039%, which was calculated based on data in Table 7 and Equation (4). For the calculation, the effects of interaction between batch, orientation, filling volume, and API were considered (E_ABC_, E_ABD_, E_ACD_, E_BCD_, and E_ABCD_).

### 3.2. Regression Analysis

Initially, an ANCOVA was performed on the long-term results in Tables S2, S4, S6, and S8 according to the ICH Q1E guideline to evaluate the variability between batches, orientations, filling volumes, and APIs. Secondly, the shelf life was predicted using a linear regression model for each product [24]. In addition, to determine the comparability between designs, the RMSE was calculated with Equation (6).

#### 3.2.1. Iron Product

**Figure 1:**
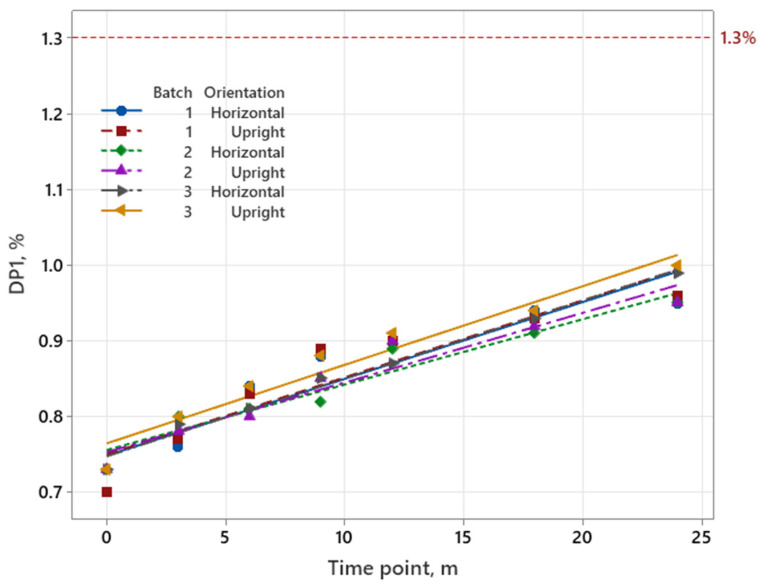
Increase in degradation products during the shelf life for three batches and two orientations for the iron product. The red line represents the specification limit for degradation products, which was 1.3%.

The performed analysis of *p*-values for slopes (batch*time) and intercepts (batch) showed that the batch-to-batch variability was higher than 0.25 [24]. Therefore, the data presented in Figure 1 for three batches and two orientations were combined into an overall estimate.

For all three designs (M1–M3), a linear regression model was used to predict the shelf life. In addition, the RMSE was also calculated to identify if the reduced designs are comparable to the full design M1. RMSE of 0.02% or lower is considered acceptable. Evaluated data are presented in Table 8.

#### 3.2.2. Pemetrexed

**Figure 2:**
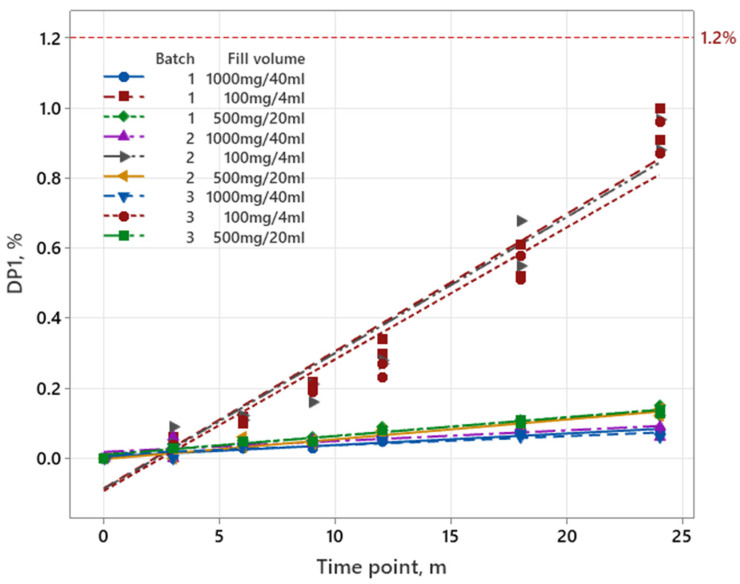
Increase in degradation products during the shelf life for three filling volumes and three batches for pemetrexed. The red line represents the specification limit for degradation products, which was 1.2%.

Data for pemetrexed were graphically analyzed, since three different filling volumes were produced. As shown in Figure 2, the increase in degradation products in the completed long-term stability study is the highest for filling volume 100 mg/4 mL.

The shelf life of the parenteral drug product is determined by filling volume 100 mg/4 mL, since the results showed a significant increase in degradation products. Consequently, further statistical analysis was performed on this filling volume. Obtained p-values for slopes (batch*time) and intercepts (batch), using the ANCOVA model, show that the batch-to-batch variability was higher than 0.25; therefore, the data were combined into an overall estimate. For designs M2 and M3, a linear regression model was used to predict the shelf life. In addition, the RMSE was calculated to identify if the reduced design M3 is comparable to the design M2. RMSE of 0.01% or lower is considered acceptable. Evaluated data are presented in Table 9.

#### 3.2.3. Sugammadex

**Figure 3:**
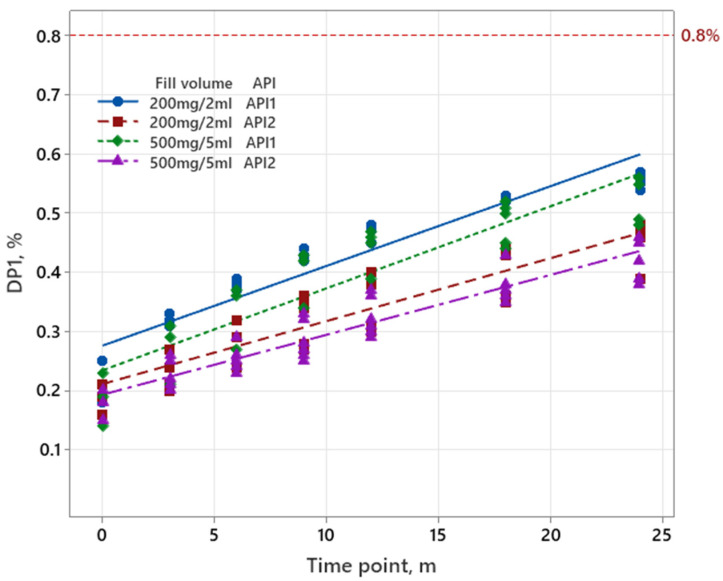
Increase in degradation products during the shelf life for two filling volumes and two API’s for sugammadex. The red line represents the specification limit for degradation products, which was 0.8%.

Data for sugammadex were graphically analyzed, since two different filling volumes were produced with two different APIs. As shown in Figure 3, the increase in degradation products in the completed long-term stability study is the highest for batches produced with API1.

The shelf life of the parenteral drug product is determined by batches produced with API1, since the results showed a significant increase in degradation products. Consequently, further statistical analysis was performed on API1. Obtained p-values for slopes (batch*time) and intercepts (batch), using the ANCOVA model, show that the batch-to-batch variability was higher than 0.25; therefore, the data were combined into an overall estimate. For reduced designs (M2–M5), a linear regression model was used to predict the shelf life. In addition, the RMSE was calculated to identify if the reduced designs (M3–M5) are comparable to the design M2. RMSE of 0.01% or lower is considered acceptable. Evaluated data are presented in Table 10.

## 4. Discussion

The stability testing of pharmaceutical products is fundamental for the determination and confirmation of the shelf life, storage conditions, safety, and efficacy of a drug product. This is a prerequisite for initial regulatory approval, as well as for approval of changes during the lifecycle of drug product and necessitates significant time and resources, which leads to increased costs. The standard approach involves extensive testing, including various time points and conditions. Despite the cost and time-saving approaches proposed within guidelines such as bracketing and matrixing, the entire long-term stability study must be conducted for all batches. One approach to stability evaluation that has not yet been implemented in the stability guidelines is the use of factorial analysis using accelerated data. The aim of our work is, therefore, to demonstrate how factorial analysis can be applied in stability evaluation and testing in conjunction with the evaluation of stability reduction level and the assessment of the reliability and effectiveness of such evaluations.

In the first step, a factorial analysis on accelerated stability data for degradation products was performed. Based on interactive effects, the significance limit was established to determine the impact of the main effects (filling volume, orientation, drug product batch, drug substance supplier). In line with the findings, a reduction of the long-term stability study was proposed. Secondly, the proposed reduced models were evaluated and compared to the full data with estimated shelf life and RMSE. This article focuses exclusively on the evaluation of stability data and the application of factorial analysis. However, if this approach would be incorporated into official ICH guidelines, the associated risks would need to be systematically identified, assessed, and mitigated through structured risk management strategies, in accordance with the principles outlined in ICH Q9.

### 4.1. Factorial Analysis for Two-Level Complete Factorial Design

In this study, three parenteral drug products were assessed using factorial designs as discussed in Section 2.4, with the number of relevant factors determining the design’s complexity. The iron product was evaluated based on two factors: batch and orientation, using a two-factor two-level factorial approach (Table 2). The evaluation of pemetrexed involved a three-factor two-level factorial approach (Table 3) with batch, orientation, and filling volume. Sugammadex was analyzed based on four factors: batch, orientation, filling volume, and API, using a four-factor two-level factorial design (Table 4). Accelerated stability data effects calculated by factorial designs are presented in Table 5, Table 6 and Table 7. To evaluate the significance of the calculated effects, a significance limit was introduced, which is 0.02% for the iron product, 0.048% for pemetrexed, and 0.039% for sugammadex. Calculated values were compared to the set significance limit to identify the most significant factor. Subsequently, a further examination of the less significant values was conducted.

For the iron product, a significant trend was observed in the calculated effects involving batch 3 based on factorial analysis of accelerated stability data. All values for the factor batch exceed the significance limit, indicating that batch 3 has the most significant impact on the stability of the parenteral drug product (see Table 5, values in bold). This finding is further confirmed by the graphical presentation of long-term stability data trend (Figure 1), where batch 3 also showed the highest increase in degradation product. Additional analysis revealed that the values for factor orientation also exceed the significance threshold for batch 3 in the upright orientation. However, its influence is about three times less than that of the factor batch. On the other hand, no significant difference was observed between the first and second batch. The analysis of the calculated values has shown that batch 3 is the least stable in an upright position (worst case). This specific combination of batch and orientation is consistent with the long-term stability data, which also indicates that this combination has the highest result for degradation products after 2 years (1.00%; Table S2).

In the case of pemetrexed, the data presented in Table 6 show that the factor with the greatest influence on stability is the filling volume (E_C_). A detailed examination of the E_C_ values revealed that the initial six experiments exhibit negative results, all above the significance limit and approximately 10-fold higher than other calculated effects. This indicates that a filling volume of 4 mL is the least stable, and the effect with the greatest impact thus determining the shelf life of the drug product. This is consistent with findings discussed in the article by Pavčnik et al. where it was found that the increase in degradation products is a consequence of the highest headspace to filling volume ratio and oxygen permeability due to the vial neck/closure combination [19]. Further analysis of values in Table 6 shows that other effects are below the significance limit, with some values for the factor orientation (E_B_) being only slightly above it. However, this occurrence appears to be random and does not indicate a specific trend. Nevertheless, a trend can be observed across the values for the factor orientation, as they all have a negative sign, indicating a higher impact of the inverted orientation. From the data analysis, it can be concluded that a filling volume of 4 mL, batch 1, is the least stable in an inverted orientation. This particular combination of filling volume, batch, and orientation is consistent with the long-term stability data, which also shows that this combination has the highest result of degradation products after 2 years (1.00%; Table S4).

The evaluation of sugammadex shows that factor API (E_D_) has the most significant impact on stability, as all values are above the significance limit. Specifically, API 1 exhibits worse stability than API 2, as evidenced by the negative trend in the values. Further analysis reveals that most of the other values are below the significance limit; however, batch 3 has the worst stability among the batches. In general, filling volume and orientation have no significant impact on the stability. However, it can be seen that the upright orientation is slightly less stable compared to the horizontal orientation. From the analysis, it can be concluded that the combination of 2 mL filling volume, batch 3, upright orientation, and API 1 exhibits the worst stability. This specific combination of filling volume, batch, orientation, and API is consistent with the long-term stability data, which also shows that this combination has the highest degradation product result after 2 years (0.57%; Table S6).

The factorial analysis performed with accelerated data allows a reduction in the number of samples required for long-term stability studies. As presented above in the examples for the iron product, pemetrexed drug and sugammadex drug products, the proposals to reduce the number of long-term stability tests are based on the calculation of effects and the evaluation of trends of the examined factors.

### 4.2. Reduction of Long-Term Stability Study and Regression Analysis for Confirmation of Reduction

In accordance with the results presented in Chapter 3.1, various reduced designs were proposed for each parenteral pharmaceutical product. For each design, both complete and reduced, a linear regression was employed to analyze long-term stability data and establish the shelf life [25]. All proposed models and results are presented in Table 8, Table 9 and Table 10.

Two reduced models were developed for the iron product, as presented in Table 8. In the M2 design, batch 2 was excluded based on the calculated effect in Table 5, as it exhibited the least impact on stability. This exclusion was made for the purposes of this study only and does not comply with ICH guideline Q1A (R2), which requires the inclusion of three batches for each filling volume in stability testing. In the M3 design, the reduction was applied to the horizontal orientation, as the data indicate that the upright orientation has the greatest impact on stability. The calculated shelf life demonstrated that the results were comparable between the full and reduced designs. It is important to note that for reduced designs, the predicted shelf life may be shorter than the actual shelf life, representing a worst-case scenario. The calculated RMSE remained below the set limit of 0.02%, further confirming the validity of the reduced models.

Based on the factorial analysis of pemetrexed, the effect of filling volume E_C_ shows the greatest impact, up to about 10-fold higher than other effects (Table 6). This observation is also confirmed by long-term stability data in Figure 2, with 4 mL of filling volume representing the worst case in terms of the increase in degradation products. Based on these findings, a reduced model M2 was developed, in which only 4 mL of filling volume was tested. Model M3 was established by further reducing M2 by excluding the upright orientation, as the data show that all results have a negative sign, indicating that the inverted orientation has the greatest impact on stability and should be included in the stability design. The calculated shelf life for M2 is the same as for M1, as the shelf life for M1 was determined based on the ICH Q1A (R2) approach. This approach states that the shelf life is calculated from the stability data of the least stable batches. In the case of pemetrexed, the least stable batch is 4 mL, as shown by the factorial analysis in Table 6 and the graphical presentation in Figure 2. Shelf life for M3 is comparable to that of M1 and M2; however, the RMSE for M3 slightly exceeds the set threshold. The obtained RMSE value is still considered acceptable as it was found that the accuracy of the model depends on the degradation kinetics [19]. All data were analyzed in line with the ICH Q1E guideline, assuming only a linear regression model [24].

For sugammadex, the factorial analysis shows the higher effect of API E_D_, which is approximately 10-fold higher than other calculated effects (Table 7). This observation is also confirmed by long-term stability data in Figure 3, with API 1 representing the worst case in terms of the increase in degradation products. Based on these findings, a reduced model M2 was developed containing only API 1 testing. Model M3 was created by further reducing M2 by excluding the 5 mL of filling volume, as the data show that most values have a negative sign, indicating that the 2 mL of filling volume have a higher impact than the 5 mL of filling volume. The M4 model was reduced by excluding the horizontal orientation of M2, as the upright orientation has a higher impact compared to the horizontal orientation (values have positive signs, Table 7). Nevertheless, all calculated effects for the factor orientation (E_B_) are below the significance limit. Finally, the M5 model was reduced by excluding the 5 mL of filling volume from M3, as the data indicate that the 2 mL of filling volume show worse stability compared to 5 mL. Shelf life for M2 was calculated in the same way as for M1 for sugammadex, considering the ICH Q1A (R2) approach. The calculated shelf life for models M2-M5 was comparable to that of M1; however, the RMSE values showed some differences. Model M4 is well below the set limit of 0.01%, while models M3 and M5 are slightly above the set limit. However, this indicates that the reduction can still be performed, as the RMSE data were evaluated assuming a linear regression model.

The factorial analysis shows that the number of combinations in long-term stability testing can be reduced for factors with higher effects, in particular, filling volume (E_C_) for pemetrexed (Table 6) and API (E_D_) for sugammadex. This is confirmed by the identical calculated shelf lives for M1 and M2, as shown in Table 9 and Table 10.

By applying a regression analysis to confirm the proposed reductions based on the factorial analysis, a 50% reduction was proposed for the iron product, from six different samples (three batches in two orientations) to three samples (three batches in one orientation). For pemetrexed, 83% reduction was proposed, from eighteen different samples (3 filling volumes, 3 batches in 2 orientations) to three samples (1 filling volume, 3 batches in one orientation), and for sugammadex reduction of 87.5%, from 24 different samples (2 filling volumes, 3 batches, 2 orientations and 2 API’s) to three samples (1 filling volume, 3 batches, 1 orientation, and 1 API). In comparison, considering the current reduced stability principles outlined in ICH Q1D, bracketing and matrixing designs, the following reductions can be achieved. A maximum of 50% reduction would be possible for all three products using a matrixing design. With a bracketing design, a 33% reduction for pemetrexed would be possible. However, bracketing is not feasible for the iron product, as there is only one filling volume, and for sugammadex with two filling volumes.

As mentioned above, ICH Q1D allows matrixing or bracketing approaches to reduce/optimize stability studies, and we have shown that the factorial analysis can also be effectively used for the same purpose. Thus, we believe that the current stability guidelines could also be extended to the application of factorial analysis for the purpose of reducing stability testing. However, this study focused exclusively on synthetic chemical entities; therefore, the conclusions drawn may not be directly applicable to other categories of pharmaceutical products, such as biologics or advanced therapy medicinal products.

## 5. Conclusions

This study demonstrates that factorial analysis of accelerated stability data can play a crucial role in reducing/optimizing long-term stability studies for parenteral pharmaceutical dosage forms for synthetic chemical entities. The factorial analysis provided key insights into the factors associated with significant influence on stability over time, which include batch, orientation, filling volume, and API. The analysis identified the worst-case scenarios and proposed a drastic reduction in the long-term stability study designs for iron product, pemetrexed, and sugammadex. Since application of factorial analysis for registration batches stability testing reduction has not yet published, to our knowledge, this work potentially paves the way for an update of current stability guidelines and for the pharmaceutical industry to optimize, accelerate, and reduce the costs associated with stability studies while still ensuring the consistent efficacy and safety of their products.

## Figures and Tables

**Table 1:** Full stability design of tested products for the proposed shelf life of 2 years.

	Iron Product	Pemetrexed	Sugammadex
Number of batches	3	3	3
Number of filling volumes	1	3	2
Number of orientations	2	2	2
Number of API	1	1	2
Sampling time (months):			
Long-term testing	0, 3, 6, 9, 12, 18, 24	0, 3, 6, 9, 12, 18, 24	0, 3, 6, 9, 12, 18, 24
Accelerated testing	0, 3, 6	0, 3, 6	0, 3, 6
Total number of samples tested	60	180	240

**Table 2:** Two-factor two-level complete factorial design for the iron product.

Experiment	A	B	AB	Answer
1	−	−	+	y_1_
2	−	+	−	y_2_
3	+	−	−	y_3_
4	+	+	+	y_4_
Effect	E_A_	E_B_	E_AB_	/

A = batch (factor), B = orientation (factor), AB = interaction between factors A and B, + or − = level in a two-level design, y = result for degradation products in % from accelerated stability study.

**Table 3:** Three-factor two-level complete factorial design for pemetrexed.

Experiment	A	B	C	AB	AC	BC	ABC	Answer
1	−	−	−	+	+	+	−	y_1_
2	−	−	+	+	−	−	+	y_2_
3	−	+	−	−	+	−	+	y_3_
4	−	+	+	−	−	+	−	y_4_
5	+	−	−	−	−	+	+	y_5_
6	+	−	+	−	+	−	−	y_6_
7	+	+	−	+	−	−	−	y_7_
8	+	+	+	+	+	+	+	y_8_
Effect	E_A_	E_B_	E_C_	E_AB_	E_AC_	E_BC_	E_ABC_	/

A = batch (factor), B = orientation (factor), C = filling volume (factor), AB/AC/BC/ABC = interaction between factors A, B, and C, + or − = level in a two-level design, y = result for degradation products in % from accelerated stability study.

**Table 4:** Four-factor two-level complete factorial design for sugammadex.

Experiment	A	B	C	D	AB	AC	AD	BC	BD	CD	ABC	ABD	ACD	BCD	ABCD	Answer
1	−	−	−	−	+	+	+	+	+	+	−	−	−	−	+	y_1_
2	−	−	−	+	+	+	−	+	−	−	−	+	+	+	−	y_2_
3	−	−	+	−	+	−	+	−	+	−	+	−	+	+	−	y_3_
4	−	−	+	+	+	−	−	−	−	+	+	+	−	−	+	y_4_
5	−	+	−	−	−	+	+	−	−	+	+	+	−	+	−	y_5_
6	−	+	−	+	−	+	−	−	+	−	+	−	+	−	+	y_6_
7	−	+	+	−	−	−	+	+	−	−	−	+	+	−	+	y_7_
8	−	+	+	+	−	−	−	+	+	+	−	−	−	+	−	y_8_
9	+	−	−	−	−	−	−	+	+	+	+	+	+	−	−	y_9_
10	+	−	−	+	−	−	+	+	−	−	+	−	−	+	+	y_10_
11	+	−	+	−	−	+	−	−	+	−	−	+		+	+	y_11_
12	+	−	+	+	−	+	+	−	−	+	−	−	+	−	−	y_12_
13	+	+	−	−	+	−	−	−	−	+	−	−	+	+	+	y_13_
14	+	+	−	+	+	−	+	−	+	−	−	+	−	−	−	y_14_
15	+	+	+	−	+	+	−	+	−	−	+	−	−	−	−	y_15_
16	+	+	+	+	+	+	+	+	+	+	+	+	+	+	+	y_16_
Effect	E_A_	E_B_	E_C_	E_D_	E_AB_	E_AC_	E_AD_	E_BC_	E_BD_	E_CD_	E_ABC_	E_ABD_	E_ACD_	E_BCD_	E_ABCD_	/

A = batch (factor), B = orientation (factor), C = filling volume (factor), D = API (factor), AB/AC/AD/BC/BD/CD/ABC/ABD/ACD/BCD/ABCD = interaction between factors A, B, C and D, + or − = level in a two-level design, y = result for degradation products in % from accelerated stability study.

**Table 8:** Calculated shelf life (months) and RMSE with long-term data for the iron product.

	Full Stability Design	Reduced Stability Design	
	M1	M2 ^A^	M3 ^B^
Number of batches	3	2	3
Number of orientations	2	2	1
Shelf life, months	52.0	49.0	49.4
RMSE, % ^C^	/	0.00462	0.00439
reduction of long-term stability study, %	/	33	50

^A^ reduction with batch 2, ^B^ reduction with horizontal orientation, ^C^ root-mean-square error (RMSE).

**Table 9:** Calculated shelf life (months) and RMSE with 24-month long-term data for pemetrexed.

	Full Stability Design	Reduced Stability Design	
	M1	M2 ^A^	M3 ^B^
Number of batches	3	3	3
Number of orientations	2	2	1
Number of filling volumes	3	1	1
Shelf life, months	31.9	31.9	29.8
RMSE, % ^C^	/	/	0.02701
reduction of long-term stability study, %	/	66	83

^A^ reduction with filling volume 500 mg/20 mL and 1000 mg/40 mL, ^B^ reduction with upright orientation for filling volume 100 mg/4 mL, ^C^ root-mean-square error (RMSE).

**Table 10:** Calculated shelf life (months) and RMSE with 24-month long-term data for sugammadex.

	Full Stability Design	Reduced Stability Design			
	M1	M2 ^A^	M3 ^B^	M4 ^C^	M5 ^D^
Number of batches	3	3	3	3	3
Number of orientations	2	2	2	1	1
Number of fill volumes	2	2	1	2	1
Number of APIs	2	1	1	1	1
Shelf life, months	37.5	37.5	36.3	37.0	35.4
RMSE, % ^E^	/	/	0.01877	0.00236	0.01703
reduction of long-term stability study, %	/	50	75	75	87.5

^A^ reduction with API2, ^B^ reduction with filling volume 500 mg/5 mL, ^C^ reduction with horizontal orientation, ^D^ reduction with filling volume 500 mg/5 mL and horizontal orientation, ^E^ root-mean-square error (RMSE).

## Data Availability

Data are contained within the article and Supplementary Materials.

## Supplementary Materials

The following supporting information can be downloaded at: https://www.mdpi.com/article/10.3390/pharmaceutics17081067/s1, Table S1: Chromatographic conditions of analytical methods for degradation products; Table S2: Long-term stability data (degradation product) for iron product; Table S3: Accelerated stability data (degradation product) for iron product; Table S4: Long-term stability data (degradation product) for three filling volumes of pemetrexed; Table S5: Accelerated stability data (degradation product) for three filling volumes of pemetrexed; Table S6: Long-term stability data (degradation product) for two filling volumes of sugammadex, API 1; Table S7: Accelerated stability data (degradation product) for two filling volumes of sugammadex, API 1; Table S8: Long-term stability data (degradation product) for two filling volumes of sugammadex, API 2; Table S9: Accelerated stability data (degradation product) for two filling volumes of sugammadex, API 2; Figure S1: Structure of examined compounds; Figure S2: Structure of monitored degradation products; Procedure S1: Example for calculating the effect E_A_, E_B_, and E_AB_.
