# Supplementary material for: Use of Factorial Designs to Reduce Stability Studies for Parenteral Drug Products: Determination of Factor Effects via Accelerated Stability Data Analysis
Source: Pharmaceutics. 2025 Aug 18;17(8):1067. doi: 10.3390/pharmaceutics17081067 (PMC12389050; doi:10.3390/pharmaceutics17081067)
Supplement: Supplementary file 1 [file pharmaceutics-17-01067-s001.zip › pharmaceutics-3789460-supplementary.pdf]

Article

Use of Factorial Designs to Reduce Stability Studies for Parenteral Drug Products: Determination of Factor Effects via Accelerated Stability Data Analysis

List of Tables:

Table S1: Chromatographic conditions of analytical methods for degradation products. ....
Table S2: Long-term stability data (degradation product) for the iron product. ....
Table S3: Accelerated stability data (degradation product) for the iron product. ....
Table S4: Long-term stability data (degradation product) for three filling volumes of pemetrexed. ....
Table S5: Accelerated stability data (degradation product) for three filling volumes of pemetrexed. ....
Table S6: Long-term stability data (degradation product) for two filling volumes of sugammadex, API 1. ....
Table S7: Accelerated stability data (degradation product) for two filling volumes of sugammadex, API 1................
Table S8: Long-term stability data (degradation product) for two filling volumes of sugammadex, API 2. ....
Table S9: Accelerated stability data (degradation product) for two filling volumes of sugammadex, API 2................

List of Figures:

Figure S1: Structure of examined compounds. ....
Figure S2: Structure of monitored degradation products. ....

List of Procedures:

Procedure S1: Example for calculating the effects E_A, E_B and E_{AB}.. ....

Table S1: Chromatographic conditions of analytical methods for degradation products.

<table>
<tr><td></td><td>iron product</td><td>pemetrexed</td><td>sugammadex</td></tr>
<tr><td></td><td>UV-VIS[1]</td><td>HPLC[2]</td><td>UHPLC[3]</td></tr>
<tr><td>Column</td><td>-</td><td>Poroshell 120 SB-C18
2.7 µm, 100 × 3.0 mm</td><td>Acquity UPLC Protein BEH C4
1.7 µm, 150 × 2.1 mm</td></tr>
<tr><td>Mobile phase</td><td>-</td><td>A: 0.02M KH_2PO_4, pH = 3.0
B: Acetonitrile</td><td>A: 0.01M NaH_2PO_4, pH = 2.5
B: Acetonitrile</td></tr>
<tr><td>Solvent</td><td>0.02M Acetate buffer solution</td><td>Purified water</td><td>Purified water: acetonitrile = 92 : 8 (V/V)</td></tr>
<tr><td>Column temperature</td><td>-</td><td>35°C</td><td>46°C</td></tr>
<tr><td>Flow rate</td><td>-</td><td>1.4 ml/min</td><td>0.33 ml/min</td></tr>
<tr><td>Gradient</td><td>-</td><td><table>
<tr><td>Time (min)</td><td>% A</td><td>% B</td></tr>
<tr><td>0</td><td>91</td><td>9</td></tr>
<tr><td>6.8</td><td>91</td><td>9</td></tr>
<tr><td>13.6</td><td>80</td><td>20</td></tr>
<tr><td>15.6</td><td>80</td><td>20</td></tr>
<tr><td>15.8</td><td>91</td><td>9</td></tr>
<tr><td>18.0</td><td>91</td><td>9</td></tr>
</table></td><td><table>
<tr><td>Time (min)</td><td>% A</td><td>% B</td></tr>
<tr><td>0</td><td>90</td><td>10</td></tr>
<tr><td>1</td><td>90</td><td>10</td></tr>
<tr><td>2</td><td>86</td><td>14</td></tr>
<tr><td>8.5</td><td>81.5</td><td>18.5</td></tr>
<tr><td>18.5</td><td>81.5</td><td>18.5</td></tr>
<tr><td>24.5</td><td>75.5</td><td>24.5</td></tr>
<tr><td>31</td><td>55</td><td>45</td></tr>
<tr><td>34</td><td>55</td><td>45</td></tr>
<tr><td>34.5</td><td>20</td><td>80</td></tr>
<tr><td>36</td><td>20</td><td>80</td></tr>
<tr><td>37</td><td>90</td><td>10</td></tr>
</table></td></tr>
<tr><td>Wavelength</td><td>592 nm</td><td>228 nm</td><td>205 nm</td></tr>
<tr><td>Injection volume</td><td>-</td><td>5 µl</td><td>2 µl</td></tr>
<tr><td>Sampler temperature</td><td>-</td><td>5°C</td><td>5°C</td></tr>
</table>

[1] Ultraviolet-visible spectroscopy, [2] High-performance liquid chromatography (HPLC), [3] Ultrahigh-performance liquid chromatography (UHPLC)

Table S2: Long-term stability data (degradation product) for the iron product.

		Time point,	iron product 1000mg/20ml
Batch	Orientation	months	DP1[1], %
1	Horizontal	0	0.70
1	Horizontal	3	0.76
1	Horizontal	6	0.84
1	Horizontal	9	0.88
1	Horizontal	12	0.90
1	Horizontal	18	0.94
1	Horizontal	24	0.95
2	Horizontal	0	0.73
2	Horizontal	3	0.80
2	Horizontal	6	0.81
2	Horizontal	9	0.82
2	Horizontal	12	0.89
2	Horizontal	18	0.91
2	Horizontal	24	0.95
3	Horizontal	0	0.73
3	Horizontal	3	0.79
3	Horizontal	6	0.81
3	Horizontal	9	0.85
3	Horizontal	12	0.87
3	Horizontal	18	0.93
3	Horizontal	24	0.99
1	Upright	0	0.70
1	Upright	3	0.77
1	Upright	6	0.83
1	Upright	9	0.89
1	Upright	12	0.90
1	Upright	18	0.93
1	Upright	24	0.96
2	Upright	0	0.73
2	Upright	3	0.78
2	Upright	6	0.80
2	Upright	9	0.85
2	Upright	12	0.90
2	Upright	18	0.92
2	Upright	24	0.95
3	Upright	0	0.73
3	Upright	3	0.80
3	Upright	6	0.84
3	Upright	9	0.88
3	Upright	12	0.91
3	Upright	18	0.94
3	Upright	24	1.00

[1] Degradation product (DP1).

Table S3: Accelerated stability data (degradation product) for the iron product.

		Time point,	iron product 1000mg/20ml
Batch	Orientation	months	DP1[1], %
1	Horizontal	0	0.70
1	Horizontal	3	0.95
1	Horizontal	6	1.09
2	Horizontal	0	0.73
2	Horizontal	3	0.95
2	Horizontal	6	1.06
3	Horizontal	0	0.73
3	Horizontal	3	1.02
3	Horizontal	6	1.16
1	Upright	0	0.70
1	Upright	3	0.95
1	Upright	6	1.06
2	Upright	0	0.73
2	Upright	3	0.93
2	Upright	6	1.07
3	Upright	0	0.73
3	Upright	3	1.01
3	Upright	6	1.28

[1] Degradation product (DP1).

Table S4: Long-term stability data (degradation product) for three filling volumes of pemetrexed.

		Time point,	pemetrexed 100mg/4ml	pemetrexed 500mg/20ml	pemetrexed 1000mg/40ml
Batch	Orientation	months	DP1[1], %	DP1[1], %	DP1[1], %
1	Inverted	0	0.00	0.00	0.00
1	Inverted	3	0.06	0.04	0.03
1	Inverted	6	0.12	0.05	0.03
1	Inverted	9	0.22	0.04	0.03
1	Inverted	12	0.34	0.09	0.05
1	Inverted	18	0.61	0.10	0.08
1	Inverted	24	1.00	0.15	0.08
2	Inverted	0	0.00	0.00	0.00
2	Inverted	3	0.09	0.00	0.00
2	Inverted	6	0.12	0.06	0.05
2	Inverted	9	0.21	0.05	0.04
2	Inverted	12	0.28	0.08	0.07
2	Inverted	18	0.68	0.10	0.09
2	Inverted	24	0.97	0.14	0.06
3	Inverted	0	0.00	0.00	0.00
3	Inverted	3	0.04	0.03	0.00
3	Inverted	6	0.11	0.05	0.03
3	Inverted	9	0.19	0.05	0.04
3	Inverted	12	0.27	0.08	0.05

3	Inverted	18	0.58	0.10	0.07
3	Inverted	24	0.96	0.14	0.06
1	Upright	0	0.00	0.00	0.00
1	Upright	3	0.05	0.03	0.00
1	Upright	6	0.10	0.04	0.03
1	Upright	9	0.21	0.06	0.03
1	Upright	12	0.30	0.08	0.05
1	Upright	18	0.52	0.11	0.07
1	Upright	24	0.91	0.12	0.07
2	Upright	0	0.00	0.00	0.00
2	Upright	3	0.04	0.00	0.06
2	Upright	6	0.11	0.03	0.04
2	Upright	9	0.16	0.04	0.05
2	Upright	12	0.27	0.07	0.06
2	Upright	18	0.55	0.10	0.08
2	Upright	24	0.88	0.12	0.09
3	Upright	0	0.00	0.00	0.00
3	Upright	3	0.04	0.03	0.03
3	Upright	6	0.10	0.05	0.03
3	Upright	9	0.21	0.05	0.04
3	Upright	12	0.23	0.08	0.05
3	Upright	18	0.51	0.11	0.06
3	Upright	24	0.87	0.13	0.06

[1] Oxidative degradation product pemetrexed S-dimer (DP1).

Table S5: Accelerated stability data (degradation product) for three filling volumes of pemetrexed.

		Time point, months	pemetrexed 100mg/4ml	pemetrexed 500mg/20ml	pemetrexed 1000mg/40ml
Batch	Orientation		DP1[1], %	DP1[1], %	DP1[1], %
1	Inverted	0	0.00	0.00	0.00
1	Inverted	3	0.33	0.06	0.04
1	Inverted	6	0.63	0.11	0.06
2	Inverted	0	0.00	0.00	0.00
2	Inverted	3	0.22	0.07	0.05
2	Inverted	6	0.47	0.12	0.06
3	Inverted	0	0.00	0.00	0.00
3	Inverted	3	0.18	0.07	0.04
3	Inverted	6	0.53	0.10	0.07
1	Upright	0	0.00	0.00	0.00
1	Upright	3	0.28	0.07	0.04
1	Upright	6	0.49	0.10	0.06
2	Upright	0	0.00	0.00	0.00
2	Upright	3	0.18	0.07	0.05
2	Upright	6	0.47	0.11	0.07
3	Upright	0	0.00	0.00	0.00
3	Upright	3	0.21	0.06	0.04
3	Upright	6	0.43	0.11	0.06

[1] Oxidative degradation product pemetrexed S-dimer (DP1).

Table S6: Long-term stability data (degradation product) for two filling volumes of sugammadex, API 1.

		Time point, months	sugammadex 200mg/2ml	sugammadex 500mg/5ml
Batch	Orientation		DP1[1], %	DP1[1], %
1	Horizontal	0	0.25	0.23
1	Horizontal	3	0.33	0.31
1	Horizontal	6	0.38	0.36
1	Horizontal	9	0.44	0.42
1	Horizontal	12	0.48	0.46
1	Horizontal	18	0.53	0.52
1	Horizontal	24	0.57	0.56
2	Horizontal	0	0.18	0.14
2	Horizontal	3	0.32	0.21
2	Horizontal	6	0.38	0.27
2	Horizontal	9	0.42	0.34
2	Horizontal	12	0.45	0.39
2	Horizontal	18	0.52	0.45
2	Horizontal	24	0.55	0.49
3	Horizontal	0	0.21	0.19
3	Horizontal	3	0.33	0.29
3	Horizontal	6	0.39	0.37
3	Horizontal	9	0.44	0.43
3	Horizontal	12	0.48	0.47
3	Horizontal	18	0.53	0.51
3	Horizontal	24	0.57	0.55
1	Upright	0	0.25	0.23
1	Upright	3	0.33	0.31
1	Upright	6	0.38	0.36
1	Upright	9	0.44	0.42
1	Upright	12	0.48	0.45
1	Upright	18	0.52	0.50
1	Upright	24	0.56	0.55
2	Upright	0	0.18	0.14
2	Upright	3	0.31	0.21
2	Upright	6	0.37	0.27
2	Upright	9	0.43	0.34
2	Upright	12	0.45	0.39
2	Upright	18	0.52	0.44
2	Upright	24	0.54	0.48
3	Upright	0	0.21	0.19
3	Upright	3	0.32	0.29
3	Upright	6	0.39	0.37
3	Upright	9	0.43	0.42
3	Upright	12	0.47	0.45
3	Upright	18	0.53	0.51
3	Upright	24	0.57	0.55

[1] Oxidative degradation product mono-S-oxo-sugammadex (DP1).

Table S7: Accelerated stability data (degradation product) for two filling volumes of sugammadex, API 1.

		Time point,	sugammadex 200mg/2ml	sugammadex 500mg/5ml
Batch	Orientation	months	DP1[1], %	DP1[1], %
1	Horizontal	0	0.25	0.23
1	Horizontal	3	0.56	0.55
1	Horizontal	6	0.69	0.71
2	Horizontal	0	0.16	0.14
2	Horizontal	3	0.46	0.46
2	Horizontal	6	0.61	0.61
3	Horizontal	0	0.20	0.19
3	Horizontal	3	0.56	0.57
3	Horizontal	6	0.71	0.73
1	Upright	0	0.25	0.23
1	Upright	3	0.56	0.55
1	Upright	6	0.70	0.71
2	Upright	0	0.16	0.14
2	Upright	3	0.47	0.45
2	Upright	6	0.62	0.61
3	Upright	0	0.20	0.19
3	Upright	3	0.57	0.56
3	Upright	6	0.73	0.74

[1] Oxidative degradation product mono-S-oxo-sugammadex (DP1).

Table S8: Long-term stability data (degradation product) for two filling volumes of sugammadex, API 2.

		Time point,	sugammadex 200mg/2ml	sugammadex 500mg/5ml
Batch	Orientation	months	DP1[1], %	DP1[1], %
1	Horizontal	0	0.19	0.18
1	Horizontal	3	0.24	0.22
1	Horizontal	6	0.29	0.24
1	Horizontal	9	0.34	0.27
1	Horizontal	12	0.38	0.31
1	Horizontal	18	0.44	0.37
1	Horizontal	24	0.47	0.42
2	Horizontal	0	0.16	0.20
2	Horizontal	3	0.20	0.26
2	Horizontal	6	0.24	0.29
2	Horizontal	9	0.27	0.32
2	Horizontal	12	0.30	0.36
2	Horizontal	18	0.35	0.43
2	Horizontal	24	0.39	0.45
3	Horizontal	0	0.21	0.15

3	Horizontal	3	0.27	0.21
3	Horizontal	6	0.32	0.26
3	Horizontal	9	0.36	0.26
3	Horizontal	12	0.40	0.30
3	Horizontal	18	0.44	0.36
3	Horizontal	24	0.47	0.39
1	Upright	0	0.19	0.18
1	Upright	3	0.24	0.22
1	Upright	6	0.29	0.25
1	Upright	9	0.34	0.28
1	Upright	12	0.38	0.32
1	Upright	18	0.44	0.38
1	Upright	24	0.48	0.42
2	Upright	0	0.16	0.20
2	Upright	3	0.21	0.25
2	Upright	6	0.24	0.29
2	Upright	9	0.28	0.33
2	Upright	12	0.31	0.37
2	Upright	18	0.36	0.43
2	Upright	24	0.39	0.46
3	Upright	0	0.21	0.15
3	Upright	3	0.27	0.20
3	Upright	6	0.32	0.23
3	Upright	9	0.35	0.25
3	Upright	12	0.38	0.29
3	Upright	18	0.43	0.35
3	Upright	24	0.46	0.38

[1] Oxidative degradation product mono-S-oxo-sugammadex (DP1).

Table S9: Accelerated stability data (degradation product) for two filling volumes of sugammadex, API 2.

		Time point, months	sugammadex 200mg/2ml	sugammadex 500mg/5ml
Batch	Orientation		DP1[1], %	DP1[1], %
1	Horizontal	0	0.19	0.18
1	Horizontal	3	0.45	0.38
1	Horizontal	6	0.60	0.53
2	Horizontal	0	0.16	0.20
2	Horizontal	3	0.36	0.44
2	Horizontal	6	0.50	0.58
3	Horizontal	0	0.21	0.15
3	Horizontal	3	0.47	0.35
3	Horizontal	6	0.61	0.49
1	Upright	0	0.19	0.18
1	Upright	3	0.45	0.38
1	Upright	6	0.61	0.53
2	Upright	0	0.16	0.20
2	Upright	3	0.36	0.43

2	Upright	6	0.51	0.57
3	Upright	0	0.21	0.15
3	Upright	3	0.47	0.35
3	Upright	6	0.62	0.48

[1] Oxidative degradation product mono-S-oxo-sugammadex (DP1).

iron product	pemetrexed	sugammadex
N/A	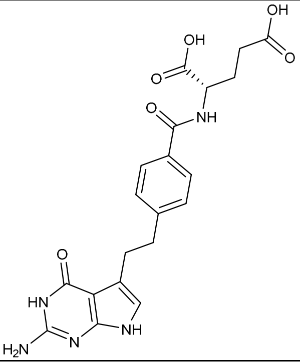	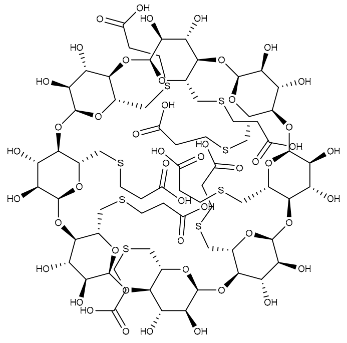

Figure S1: Structure of examined compounds.

Degradation product for iron product	pemetrexed S-dimer	mono-S-oxo-sugammadex
N/A	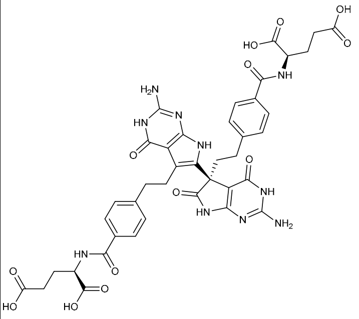	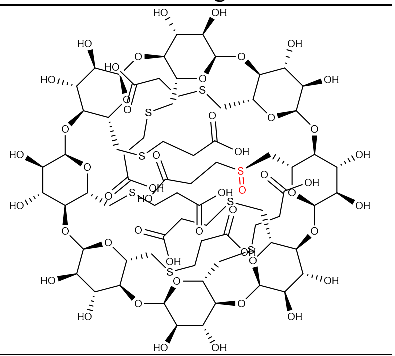

Figure S2: Structure of monitored degradation products. Source: ChemDraw® 23.1.2.7 (Revvity Signals Software, Inc., Waltham, Massachusetts, U.S.).

Procedure S1: Example for calculating the effect E_A, E_B and E_{AB} for batches 1 and 3, from Table 5 in the Article.

1. Two-factor two-level complete factorial design for the iron product.

Experiment	A	B	AB	Answer
1	−	−	+	y_1
2	−	+	−	y_2
3	+	−	−	y_3
4	+	+	+	y_4
Effect	E_A	E_B	E_{AB}	/

2. Two-factor two-level complete factorial design for the iron product filled with data.

Experiment	A = batch	B = orientation	AB = interaction	Answer = % of degradation product at 6 months accelerated stability study*
1	−(1)	−(→)	+ (1, →)	$y_1 = 1.09$
2	−(1)	+ (↑)	−(1, ↑)	$y_2 = 1.06$
3	+ (3)	−(→)	−(3, →)	$y_3 = 1.16$
4	+ (3)	+ (↑)	+ (3, ↑)	$y_4 = 1.28$
Effect	E_A	E_B	E_{AB}	/

*Stability data from Table S3.

3. Equation for calculating the effect for factor A, E_A:

$$Effect\ A = (-y_1 - y_2 + y_3 + y_4)/2$$

4. Calculation of effect for factor A, E_A:

$$Effect\ A = (-1.09 - 1.06 + 1.16 + 1.28)/2 = 0.145$$

5. Equation for calculating the effect for factor B, E_B:

$$Effect\ B = (-y_1 + y_2 - y_3 + y_4)/2$$

6. Calculation of effect for factor B, E_B:

$$Effect\ B = (-1.09 + 1.06 - 1.16 + 1.28)/2 = 0.045$$

7. Equation for calculating the effect for interaction AB, E_{AB}:

$$Effect\ AB = (+y_1 - y_2 - y_3 + y_4)/2$$

8. Calculation of effect for interaction AB, E_{AB}:

$$Effect\ AB = (+1.09 - 1.06 - 1.16 + 1.28)/2 = 0.075$$
